# Antibiograms of field and hospital acquired equine neonatal bacterial fluid cultures in the Midwestern United States: 149 samples (2007‐2018)

**DOI:** 10.1111/jvim.16671

**Published:** 2023-04-07

**Authors:** Lauren C. Bookbinder, Rinosh Mani, Elizabeth A. Carr

**Affiliations:** ^1^ College of Veterinary Medicine Michigan State University East Lansing Michigan USA

**Keywords:** anaerobic bacteria, antimicrobial resistance, bacterial culture, gram‐negative bacteria, gram‐positive bacteria, microbiology, sepsis

## Abstract

**Background:**

Contemporary data reflecting local pathogens and their antibiograms is necessary to select empirical antimicrobial therapy for equine neonates.

**Hypothesis/Objectives:**

Describe bacterial isolates associated with equine neonatal infection and their antibiograms in the Midwestern United States. An increase in gram‐positive infection and antibiotic resistance compared to previous literature was expected.

**Animals:**

Data from 149 fluid samples from 133 foals <30 days of age submitted for bacterial culture between January 2007 and December 2018.

**Methods:**

A retrospective evaluation of equine neonatal fluid cultures was performed. Fluid submission type, bacterial culture and antibiogram, empirical antibiotic treatment, and foal outcome was included. Isolate susceptibility to individual antimicrobials and combination protocols relevant to equine practice were recorded. The effect of recorded variables on foal survival was evaluated using Fisher's exact or chi‐squared tests.

**Results:**

Ninety bacterial isolates (78 aerobes and 12 anaerobes) were identified and gram‐positive organisms predominated (n = 50/90, 56%). Greater than 70% of aerobic isolates were susceptible to ampicillin, ceftiofur, chloramphenicol, trimethoprim/sulfamethoxazole, and all penicillin/aminopenicillin and aminoglycoside combinations. Seventy‐seven (n = 81/105) percent of foals survived. Survival was associated with a negative fluid culture and was not associated with empirical antimicrobial choice.

**Conclusions and Clinical Importance:**

Gram positive and anaerobic isolates associated with equine neonatal fluid cultures exceed that of previous reports. Historical empirical antimicrobial choices for equine neonatal infection in the Midwestern United States are supported by local antibiogram results.

**Abbreviations**
CLSI: Clinical and Laboratory Standards Institute
MDR: multiple drug resistance
MIC: minimum inhibitory concentration

## 1 INTRODUCTION

Equine neonatal sepsis is the combination of demonstrable infection and clinical evidence of systemic inflammation which includes at least 3 of the following: elevated rectal temperature, tachycardia, tachypnea, abnormal leukocyte count (leukocytosis, leukopenia, or band neutrophilia), venous hyperlactatemia, or venous hypoglycemia.^1^ Despite advancements in equine critical care, neonatal sepsis remains a frustrating and important cause of mortality.^2^, ^3^, ^4^, ^5^, ^6^, ^7^ Survival from sepsis is associated with prompt administration of an appropriate antimicrobial^8^, ^9^; however, bacterial culture and sensitivity is time consuming and lacks both sensitivity and specificity.^6^, ^10^, ^11^, ^12^ As a result antimicrobials are often empirically chosen, and this is complicated by temporal and regional shifts in relevant bacterial pathogens and their antibiograms. Prescribing veterinarians require a clear understanding of these shifts to make rational antimicrobial choices for foals in their care, but regional specific data are largely unavailable. The objective of this study was to document the bacterial culture and susceptibility results from fluid samples collected from foals <30 days of age and submitted to the Michigan State University Veterinary Diagnostic Laboratory over a 10‐year period to guide rational empirical antimicrobial choices for contemporary practitioners. An increase in gram‐positive infection and antibiotic resistance consistent with contemporary trends was expected.

## 2 MATERIALS AND METHODS

Bacterial fluid cultures including blood, synovial, cerebrospinal, and peritoneal samples from foals <30 days of age submitted to the Michigan State University Veterinary Diagnostic Lab from January 2007 to December 2018 were reviewed. Field and hospital acquired samples were included. When multiple fluid cultures were submitted from a single foal, each culture was evaluated independently and comparisons between results described.

Hospital submitted blood cultures were collected aseptically at the time of intravenous jugular catheter placement. Briefly, 7 to 8 mL of blood was sterilely collected and used to inoculate commercially available aerobic (VersaTREK Redox 1, 40 mL with stir bar, Trek Diagnostic Systems, Cleveland, Ohio) and anaerobic (VersaTREK Redox 2, 40 mL with stir bar, Trek Diagnostic Systems, Cleveland, Ohio) fluid cultures. Hospital submitted nonblood cultures were sterilely collected and the sample was either inoculated into fluid culture broth as above or placed in a vacutainer tube without additive based on sample volume. Aerobic and anaerobic media inoculation and culture submission are routine for the reporting hospital; however, aerobic culture was prioritized if sample volume was small. Sample collection technique was not available for field submissions. The percentage of submissions with inoculation of both aerobic and anaerobic media was not determined.

In vitro susceptibility to antimicrobials was recorded for aerobic isolates using broth microdilution method. Briefly, 10 μL of 0.5 McFarland suspension of the bacteria in sterile water was inoculated to 10 mL of cation‐adjusted Mueller‐Hinton broth. Fifty microliters of this bacterial suspension were added to each well of an equine specific antimicrobial sensitivity testing plate (Equin1F Sensititre, ThermoFisher, Waltham, Massachusetts) and the plate was incubated at 35°C for 16 to 18 hours. After incubation the minimum inhibitory concentration (MIC) for each antimicrobial was manually read and recorded. Antimicrobials were included for analysis if they were considered practical for equine neonatal use and susceptibility data was reported for at least 50% of all bacterial isolates obtained. Amikacin, ampicillin, cefazolin, ceftiofur, chloramphenicol, enrofloxacin, gentamicin, penicillin, tetracycline, and trimethoprim/sulfamethoxazole were included. Bacterial susceptibility was also reported for antimicrobial combinations common to equine practice and included amikacin/ampicillin, amikacin/penicillin, gentamicin/ampicillin, and gentamicin/penicillin. Susceptibility data was reported in ordinance with Clinical and Laboratory Standards Institute (CLSI) established breakpoints. Multiple drug resistant (MDR) isolates were defined by resistance to 3 or more evaluated antimicrobials.^11^

Case data including age, breed, and sex were recorded for all foals, and antimicrobial therapy and survival data were reported for all hospitalized foals. Empirical antimicrobial therapy was defined as the first antimicrobial administered to the foal. This therapy was categorized as “appropriate” if the antimicrobial demonstrated in vitro efficacy against organism(s) isolated from the foal's first fluid culture submission(s). Empirical antimicrobial efficacy was only evaluated if: the initial fluid sample yielded positive growth, an antibiogram was available for the recovered isolate(s) and the specific empirical antimicrobial used was recorded. If serial fluid cultures were submitted, changes in the isolated bacterial agents and their antibiograms were reported but not used to define empirical antimicrobial efficacy. Survival was defined as survival to hospital discharge and was not considered for field cases. Clinical and biochemical case data required to identify systemic inflammation was not evaluated, and enrolled foals with positive fluid cultures could not be defined as septic.

Foal age was tested for normality using a Shapiro test, and year of submission was assessed for uniform distribution. Categorical data included submission type (hospital or field), fluid type, bacterial isolate, antimicrobial sensitivity, empirical antimicrobial efficacy, and survival. Relationships between these categorical variables were evaluated using a Fisher's exact (2 variables) or chi‐squared (>2 variables) tests.

## 3 RESULTS

One hundred forty‐nine fluid cultures were performed on 133 foals <30 days of age. This included 124 hospital acquired fluid cultures (n = 108 foals) and 25 field acquired fluid cultures (n = 25 foals). Seventy‐six males and 49 females were enrolled and sex was not recorded in 8 foals. Breeds were representative of the local population and included quarter horse/paint, other/mixed, Arabian, warmblood, standardbred, and thoroughbred most commonly.

Average age at the time of fluid culture submission was 6.6 days (range, 0‐29), and age at submission differed between blood and synovial fluid samples (4.6 ± 3 and 10.8 ± 9 days respectively, *P* = .02). Sixty‐six percent (n = 98/149) of fluid submissions were blood and 34% (n = 50/149) were synovial fluid; 1 cerebrospinal fluid culture was submitted. Synovial fluid samples were overrepresented in field submissions (*P* = .0001). Fluid culture submissions were uniform across the study time period.

**FIGURE 1:**
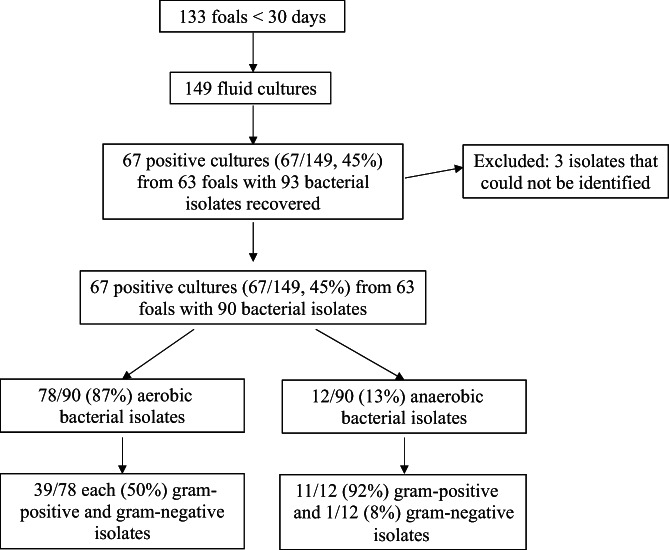
Flowchart of enrolled foals and bacterial fluid culture results. Isolates are first categorized based on aerobic or anaerobic metabolism then by gram status.

Sixty‐seven individual fluid cultures (n = 67/149, 45.0%) from 63 (n = 63/133, 47.4%) foals yielded 93 bacterial organisms. Of these organisms 3 could not be accurately identified, leaving 90 isolates for analysis (Figure 1). Forty‐four percent of blood cultures (n = 43/98) and 48% (n = 24/50) of synovial cultures yielded at least 1 isolate, and the single cerebrospinal fluid culture yielded no growth. Polymicrobial growth occurred in <10% of cultures (n = 13/149, 8.7%).

Seventy‐eight (n = 78/90, 87%) aerobic and 12 (n = 12/90, 13%) anaerobic organisms were isolated, and gram‐positive organisms predominated (n = 50/90, 56%). Specifically, 50% (n = 39/78) of aerobes and 92% (n = 11/12) of anaerobes were gram positive (Figure 1). *Escherichia coli* was the most common isolate (n = 14/90, 16%) followed by *Actinobacillus* spp. (n = 13/90, 14%), *Staphylococcus* spp. (n = 12/90, 13%), *Streptococcus* sp. (n = 11/90, 12%) and *Enterococcus* sp. (n = 9/90, 10%). Table 1 outlines the percent, number, and fluid type for each genus isolated.

**TABLE 1:** Percent of total isolates and number of each isolate genus recovered from foal fluid culture submission.

Isolate genus	Percent of total and (number) of each genus	Fluid submission type
*Escherichia*	16% (14/90)	10 blood, 4 synovial
*Actinobacillus*	14% (13/90)	7 blood, 6 synovial
*Staphylococcus*	13% (12/90)	7 blood, 5 synovial
*Streptococcus*	12% (11/90)	7 blood, 4 synovial
*Enterococcus*	10% (9/90)	8 blood, 1 synovial
*Clostridium*	7% (6/90)	5 blood, 1 synovial
*Bacillus*	4% (4/90)	4 blood
*Enterobacter*	3% (3/90)	2 blood, 1 synovial
*Acinetobacter*	2% (2/90)	2 synovial
*Corynebacterium*	2% (2/90)	1 blood, 1 synovial
*Klebsiella*	2% (2/90)	1 blood, 1 synovial
*Salmonella*	2% (2/90)	2 synovial
*Citrobacter*	1% (1/90)	1 synovial
*Cutibacterium*	1% (1/90)	1 blood
*Fusobacterium*	1% (1/90)	1 synovial
*Lactobacillus*	1% (1/90)	1 blood
*Micrococcus*	1% (1/90)	1 blood
*Moraxella*	1% (1/90)	1 blood
*Providencia*	1% (1/90)	1 synovial
*Rothia*	1% (1/90)	1 blood
*Streptomyces*	1% (1/90)	1 blood

*Note*: The number of blood or synovial fluid submissions associated with isolate recovery are listed.

Ten foals had multiple fluid cultures performed including blood and synovial fluid cultures (n = 6), multiple synovial fluid cultures (n = 2) repeat blood cultures (n = 1) and blood and CSF cultures (n = 1). For no foal were all isolates identical in repeat samples; for 6 foals the same bacteria were isolated from multiple cultures, but in each of these 6 foals disparate isolates were also recovered.

### 3.1 Susceptibility

Susceptibility profiles for all were available for 78% of aerobic isolates (n = 61/78), and included 67% (n = 26/39) of gram‐positive and 90% (n = 35/39) of gram‐negative aerobes. Chloramphenicol was the most effective in vitro antimicrobial across all aerobes, followed by ceftiofur. Ninety‐two percent (n = 56/61) of all aerobes were susceptible to chloramphenicol including 96% (n = 25/26) of gram‐positive and 89% (n = 31/35) of gram‐negative aerobes. Eighty‐five percent (n = 52/61) of all aerobes were susceptible to ceftiofur, including 73% (n = 19/26) of gram‐positive and 94% (n = 33/35) of gram‐negative aerobes. Greater than or equal to 70% of bacterial isolates were susceptible to ampicillin (n = 46/61, 75%), trimethoprim/sulfamethoxazole (n = 47/61, 77%), and all combination protocols (Tables 2 and 3). Combination protocols were equivalent across all isolates (*X* ^2^ [3, N = 61] = 6, *P* = .11; gram positive: *X* ^2^ [3, N = 26] = 1.6, *P* = .65; gram negative: *X* ^2^ [3, N = 35] = 5.4, *P* = .14; Table 3). Isolate sensitivities to individual (Table 2) and combination antimicrobials (Table 3) are reported for all, gram‐positive, and gram‐negative aerobes, and individual genera making up ≥10% of aerobic isolates.

**TABLE 2:** Antibiograms of aerobic bacterial isolates for clinically relevant individual antimicrobials.

	Amikacin %S, S/NS	Ampicillin %S, S/NS	Cefazolin %S, S/NS	Ceftiofur %S, S/NS	Chloramphenicol %S, S/NS	Enrofloxacin %S, S/NS	Gentamicin %S, S/NS	Penicillin %S, S/NS	Tetracycline %S, S/NS	TMS %S, S/NS
All aerobes (n = 78)	57%, 35/61	75%, 46/61	67%, 28/42	85%. 52/61	92%, 56/61	68%, 41/60	54%, 33/61	52%, 23/44	68.9%, 42/61	77%, 47/61
Gram‐negative aerobes (n = 39)	75%, 27/36	69%, 25/36	65%, 13/20	94%, 33/35	89%, 31/25	85%, 29/34	69%, 24/35	21%, 4/19	77%, 27/35	83%, 29/35
Gram‐positive aerobes (n = 39)	32%, 8/25	84%, 21/25	68%, 15/22	73%, 19/26	96%, 25/26	46%, 12/26	35%, 9/26	76%, 19/25	58%, 15/26	69%, 18/26
*Escherichia coli* (n = 14)	91.7%, 11/12	92%, 11/12	83%, 5/6	92%, 11/12	92%, 11/12	92%, 11/12	92%, 11/12	17%, 1/6	83%, 10/12	91.7%, 11/12
*Actinobacillus* spp. (n = 13)	62%, 8/13	100%, 13/13	100%, 7/7	100%, 13/13	100%, 13/13	77%, 10/13	54%, 7/13	43%, 3/7	100%, 13/13	69.2%, 9/13
*Staphylococcus* spp. (n = 12)	100%, 7/7	75%, 6/8	100%, 7/7	100%, 8/8	100%, 8/8	100%, 8/8	88%, 7/8	75%, 6/8	63%, 5/8	100%, 8/8
*Streptococcus* spp. (n = 11)	0%, 0/11	89%, 8/9	0%, 0/8	100%, 9/9	100%, 9/9	22%, 2/9	11%, 1/9	78%, 7/9	44%, 4/9	89%, 8/9
*Enterococcus* spp. (n = 9)	0%, 0/8	86%, 6/7	0%, 0/7	13%, 1/8	88%, 7/8	25%, 2/8	0%, 0/8	71%, 7/9	63%, 5/8	13%, 1/8

*Note*: Individual species or genera were included if they represented at least 10% of recovered isolates. Denominators are variable across rows because sensitivity to each antimicrobial was not uniformly evaluated within each isolate category.

Abbreviations: NS, not‐susceptible; S, susceptible; TMS, trimethoprim sulfamethoxazole.

**TABLE 3:** Antibiograms of aerobic bacterial isolates for clinically relevant antimicrobial combinations.

	Amikacin/Ampicillin %, S/NS	Amikacin/Penicillin %, S/NS	Ampicillin/Gentamicin %, S/NS	Penicillin/Gentamicin %, S/NS
All aerobes (n = 78)	90%, 55/61	80%, 49/61	89%, 54/61	77%, 47/61
Gram‐negative aerobes (n = 39)	91%, 32/35	77%, 27/35	86%, 30/35	71%, 25/35
Gram‐positive aerobes (n = 39)	89%, 23/26	81%, 21/26	92%, 24/26	85%, 22/26
*Escherichia coli* (n = 14)	100%, 12/12	100%, 12/12	100%, 12/12	100%, 12/12
*Actinobacillus* spp. (n = 13)	100%, 13/13	62%, 8/13	100%, 13/13	62%, 8/13
*Staphylococcus* spp. (n = 12)	100%, 8/8	88%, 7/8	100%, 8/8	88%, 7/8
*Streptococcus* spp. (n = 11)	89%, 8/9	78%, 7/9	100%, 9/9	89%, 8/9
*Enterococcus* spp. (n = 9)	75%, 6/8	75%, 6/8	75%, 6/8	63%, 5/8

*Note*: Individual species or genera were included if they represented at least 10% of recovered isolates. Denominators are variable across rows because sensitivity to each antimicrobial was not uniformly evaluated within each isolate category.

Abbreviations: NS, not‐susceptible; S, susceptible; TMS, trimethoprim sulfamethoxazole.

Thirty‐four percent (n = 20/61) of isolates demonstrated resistance to >3 relevant antimicrobials and were considered MDR. This included *Enterococcus* spp. (n = 8/8, 100%), *Streptococcus* spp. (4/11, 36%), *Enterobacter* spp. (n = 3/3, 100%), *Salmonella* sp. (n = 2/2, 100%), *Actinobacillus* sp. (n = 1/13, 8%), *Citrobacter freundii* (n = 1/1, 100%), *E. coli* (n = 1/14, 8%) and *Staphylococcus xylosus* (n = 1/12, 8%). Fifty percent (n = 13/26) of gram‐positive and 22% (n = 8/35) of gram‐negative aerobes were MDR. Greater than 70% of MDR isolates were sensitive to chloramphenicol (71%, n = 15/21) and the combination of amikacin/ampicillin (71%, n = 15/21) but all combination protocols remained statistically noninferior (*X* ^2^ [4, n = 21] = 2.0, *P* = .57).

### 3.2 Survival

Survival data was available for 105 of 108 hospital admitted foals and was unavailable for all foals evaluated in the field. Seventy‐seven percent (n = 81/105) of foals survived to hospital discharge. Foals with growth of at least 1 bacterial organism from at least 1 fluid culture were less likely to survive (68%, n = 32/47) compared to foals with negative fluid cultures (85%, n = 49/58; *P* = .05). Of the 63 foals with positive initial fluid culture results, empirical antimicrobial therapy was not reported for 8 field cases, and complete antibiogram data for recovered isolates was not reported for 30 hospitalized cases which most commonly this occurred when an anaerobe of laboratory‐defined contaminant organism was isolated.

As a result, empirical antimicrobial therapy and complete antibiogram data was available for only 25 foals with positive initial fluid culture results and 64% (n = 16/25) of these foals survived. Empirical initial antimicrobial therapy was considered appropriate based on subsequent antibiogram data in 75% (n = 19/25) of foals. Four of the remaining 6 foals received ceftiofur sodium at extra‐label doses (5‐10 mg/kg IV Q6‐12). Sixty‐eight percent of foals that received appropriate initial antimicrobials (n = 13/19) and 50% of foals that did not receive appropriate empirical antimicrobials (n = 3/6) survived (*P* = .41). All foals for which empirical antimicrobial choice was not appropriate had MDR bacteria isolated from blood culture.

## 4 DISCUSSION

This regionally specific report describes the contemporary bacterial agents isolated from equine neonatal fluid samples and their antibiograms in the Midwestern United States. Results are consistent with the authors hypothesis and support the emerging trend for gram‐positive infection in veterinary^6^, ^7^, ^10^, ^12^, ^13^, ^14^ and human^15^ literature. Specifically, gram‐positive anaerobes were more frequently isolated compared to previous reports.^12^, ^13^, ^16^, ^17^ Greater than 70% of isolates demonstrated in vitro susceptibility to ampicillin, ceftiofur, chloramphenicol, trimethoprim sulfamethoxazole, and all antimicrobial combination protocols.^12^, ^14^, ^17^, ^18^

Anaerobic bacteria represented 13% percent of isolates (n = 12/90), exceeding that of previous reports (1.2%‐6.3%).^6^, ^12^, ^13^, ^16^, ^17^ Anaerobic isolation efforts from foal fluid samples are routine at the reporting hospital, but not universally standard practice.^4^, ^6^, ^7^, ^10^, ^12^, ^17^ This likely contributed to the prevalence of anaerobic infection in this report compared to existing literature; however, a true emergence of anaerobic infection in foals must also be considered. Importantly, this study did not quantify the percent of submissions for which both aerobic and anaerobic media were inoculated. Though dual submission is standard for the reporting hospital, when sample volume or owner finances prevent culture of both media, aerobic culture is prioritized. Thus, anaerobic isolates likely remain underrepresented, and results of this study support routine anaerobic fluid culture from foals in both hospital and field settings. Anaerobes are responsible for approximately 10% of human bacteremic episodes, and anaerobic bacteremia is associated with neonatal age, necrotizing enterocolitis, and death,^19^ particularly if appropriate antimicrobial therapy is delayed.^20^ Specific anaerobic culture efforts are recommended for septic humans despite increased expense and redundancy in isolate recovery.^21^, ^22^

Fluid submission type was associated with isolate gram status. Gram‐negative isolates were more common in synovial samples, and gram‐positive isolates were more common in blood samples. However, this difference was largely attributed to gram‐positive anaerobes, and their recovery be related to systematic differences in the categories of fluid collected in this study. Firstly, collected synovial fluid volume might be insufficient to inoculate both anaerobic and aerobic media, with preference given to aerobic inoculation. Secondly, synovial fluid samples were overrepresented in field submissions and anaerobic media inoculation of foal fluid samples might not be routine for field practitioners, although this was not quantified. It is, however, also possible that there are pathophysiologic differences in infection based on gram status in foals as is seen in human medicine^23^ and has been suggested in foals.^16^ Supportively, Sanchez et al^17^ identified an association between gram‐negative sepsis and osteomyelitis in foals, whereas Hackett et al^11^ demonstrated transient and clinically‐silent gram‐positive bacteremia in foals less than 24 hours of age.

Unique bacterial isolates were cultured from different fluid samples in 7/10 foals. This could be a consequence of sample contamination, or might reflect the dynamic nature of bacterial infection in foals. A recent study comparing serial blood cultures in equine neonates found that approximately 20% of foals had novel bacterial growth after ≥48 hours of hospitalization compared to admission,^24^ and there is transient gram‐positive bacteremia in 4 otherwise healthy equine neonates less than 24 hours of age.^11^ Either novel infection or transient, clinically relevant bacteremia could contribute to disparate serial fluid culture results in foals and further investigation is warranted to support rational antimicrobial choices through a dynamic septic process.

Greater than 70% of recovered isolates were susceptible to ampicillin, ceftiofur, chloramphenicol, trimethoprim sulfamethoxazole, and all antimicrobial combination protocols. There were no significant differences in susceptibility across combination protocols. Chloramphenicol was the most effective individual antimicrobial, with 92% of isolated bacteria demonstrating in vitro sensitivity. However, its use in equine neonates faces pharmacologic and practical limitations. In adult horses repeat oral dosing of chloramphenicol fails to achieve CLSI MIC targets for >50% of the conventional dosing interval.^25^ Limited pharmacokinetic data is available on oral administration of chloramphenicol in foals, but the data available suggest mean peak plasma concentrations are highly variable among individuals and unlikely to consistently reach CLSI breakpoints.^26^ This could be related to reduced bioavailability associated with gastrointestinal dysfunction in sick foals,^27^ though this finding is inconsistent.^24^ Nonetheless, without supportive pharmacokinetic evidence, concerns regarding achievable plasma concentrations can dissuade the use of chloramphenicol in foals despite in vitro susceptibility support. Additionally, the small but serious risk of aplastic anemia in humans exposed to chloramphenicol during administration must be considered before prescription. Oral dosing is challenging in foals and amplifies this risk.

In this study, 85% of isolates were susceptible to ceftiofur at the CLSI MIC breakpoint of ≤2 μg/mL. This exceeds isolate susceptibility in geographically distinct studies.^12^, ^14^, ^17^, ^18^ There was no statistical difference between isolate susceptibility to ceftiofur compared to penicillin or aminopenicillin/aminoglycoside combination protocols. This suggests that in the study region ceftiofur could be a useful monotherapeutic antimicrobial for foals, but is not superior to more traditional combination protocols. Ceftiofur sodium (Naxcel, Zoetis, Troy Hills, New Jersey) is an attractive antimicrobial for foals because it is broad spectrum and the dose can be adjusted based on culture results and clinical progression. Extra‐label ceftiofur sodium (Naxcel, Zoetis, Troy Hills, New Jersey) dosing regimens are reported in foals and document steady state plasma ceftiofur metabolite (desfuroylceftiofur acetate) concentrations far exceeding 2 μg/mL (>15 μg/mL [10 mg/kg ceftiofur sodium IV q 6]^28^; >8 μg/mL [ceftiofur sodium 2.2 mg/kg IV] followed by 12 μg/kg/min constant rate infusion^29^). Thus, in vivo ceftiofur susceptibility is likely underrepresented by current CLSI breakpoints when extra‐label dosing regimens are used. Expected plasma concentrations of desfuroylceftiofur acetate for a wide range of dosing regimens has been reported and can be used to guide dosing decisions based on target MIC for isolated pathogens. Additionally, ceftiofur can be practically continued as needed following hospital discharge using ceftiofur crystalline free‐acid (Excede, Zoetis, Troy Hills, New Jersey) at 13.2 mg/kg SQ Q48,^30^, ^31^ which avoids a change in antimicrobial class to facilitate on‐farm administration. Importantly, ceftiofur is classified as a Critically Important Antimicrobial by the World Health Organization. Responsible antimicrobial stewardship should discourage ceftiofur sodium use if practical and effective alternatives exist.

Despite increased isolate sensitivity to ceftiofur compared to contemporary reports, other antibiogram results of this study support emerging trends in antimicrobial resistance.^12^, ^14^, ^18^ Only 62% of *Actinobacillus* spp. were susceptible to combination protocols containing penicillin, whereas 100% were susceptible to protocols containing ampicillin. Sixty‐nine percent of gram‐negative isolates were susceptible to gentamicin and 75% to amikacin, supportive of emerging aminoglycoside resistance.^14^, ^17^, ^18^ Thirty‐four percent of isolates were MDR, consistent with contemporary reports.^14^ These isolates were most commonly gram positive, and demonstrated acceptable (>70%) in vitro sensitivity to chloramphenicol and the combination of amikacin/ampicillin. Temporal changes to local isolate recovery^7^, ^17^ and antibiograms^17^ have been reported. The number of positive fluid cultures with complete antibiogram over the short study period was insufficient for this analysis.

Seventy‐seven percent of foals survived to hospital discharge and survival was associated with a negative fluid culture as reported.^2^, ^4^, ^10^, ^12^, ^16^, ^18^ In contrast to previous reports, survival was not associated with polymicrobial infection^16^ or in vitro susceptibility of the bacterial isolate to empirical antimicrobial choices.^9^ Inappropriate empirical antimicrobial therapy was identified in 6 out of 24 foals; however, 4/6 of these foals received extra‐label ceftiofur sodium at 5 to 10 mg/kg IV every 6 to 12 hours, and in vivo ceftiofur concentrations likely exceeded MIC breakpoints. The association between in vitro efficacy of an initial antimicrobial choice and survival in septic animals is inconsistent. A 1.5‐fold increased risk of death is reported when septic foals were treated with empirical antimicrobials that were ultimately deemed inappropriate.^9^ However, a recent study in septic dogs found no association between survival and empirical antimicrobial treatment.^32^ Other animal or treatment factors, and particularly the differences between in vivo and in vitro antimicrobial susceptibility likely contribute to these inconsistencies. Importantly, survival analysis was not the primary aim of this study, and the association between survival and empirical antimicrobial choice was only evaluated in 25 hospitalized foals. Notably, euthanasia due to financial constraints was not identified and undoubtedly influenced outcomes, particularly because this study period included an economic recession.

This study faces several limitations related to both sample size, retrospective data collection, and inclusion of both hospital and field‐based submissions. Incomplete and small data sets prevented some useful observations, such as comparing culture and antibiogram results across the study period, or between hospital and field submissions, which are reported.^7^, ^17^, ^33^ Similarly, clinical and biochemical data were not reported for included cases, and without this data sepsis could not be defined in our study population.^1^ Differences between hospital and field derived fluid samples also influenced results. Field submissions were collected from older foals and were more likely to be synovial compared to hospital acquired samples. Presumably this is because field cases were more advanced in their disease process and more commonly presented with signs of infectious orthopedic disease compared to systemic infection. Though these differences challenge direct comparisons between the 2 populations, they also highlight missed opportunities for field practitioners to potentially identify and treat bacteremia in younger sick foals. Many field practitioners are faced with financial and practical barriers to referral, and early blood culture of sick foals can support informed antimicrobial choices that expand the regional understanding of bacteremia in the nonhospitalized, sick foals.

Fluid cultures are inherently susceptible to both false negative and false positive results, which might under‐ or overestimate the prevalence of clinically relevant isolates. Until more precise bacteriologic identification methods are routinely available, all reports of equine neonatal infection are liable to these inaccuracies. Polymicrobial infection or disparate serial fluid culture results reported in this study might represent sample contamination; however, both findings are also reported clinically^24^ and were therefore included for analysis.

## CONFLICT OF INTEREST DECLARATION

Authors declare no conflict of interest.

## OFF‐LABEL ANTIMICROBIAL DECLARATION

Ceftiofur sodium used off‐label.

## INSTITUTIONAL ANIMAL CARE AND USE COMMITTEE (IACUC) OR OTHER APPROVAL DECLARATION

Authors declare no IACUC or other approval was needed.

## HUMAN ETHICS APPROVAL DECLARATION

Authors declare human ethics approval was not needed for this study.
